# The Impact of Brexit on UK Food Standards and Food Security: Perspectives on the Repositioning of Neoliberal Food Policy

**DOI:** 10.3390/foods14091474

**Published:** 2025-04-23

**Authors:** Sophia Lingham, Aleksandra Kowalska, Jarosław Kowalski, Damian Maye, Louise Manning

**Affiliations:** 1 Department of Agriculture, Royal Agricultural University, Stroud Road, Cirencester GL7 6JS, UK; sophia.lingham@student.rau.ac.uk; 2 Institute of Economics and Finance, Maria Curie-Sklodowska University, pl. Marii Curie-Sklodowskiej 5, 20-031 Lublin, Poland; aleksandra.kowalska@mail.umcs.pl; 3 Institute of Law Sciences, Maria Curie-Sklodowska University, pl. Marii Curie-Sklodowskiej 5, 20-031 Lublin, Poland; jaroslaw.kowalski@mail.umcs.pl; 4 Countryside and Community Research Institute, University of Gloucestershire, Francis Close Hall Campus, Swindon Road, Cheltenham GL50 4AZ, UK; dmaye@glos.ac.uk; 5 The Lincoln Institute of Agri-Food Technology, University of Lincoln, Lincoln LN2 2DP, UK

**Keywords:** food, Brexit, neoliberal, food trade, regulatory environment, food standards, food security, neo-developmentalism

## Abstract

Brexit, the exiting of the United Kingdom (UK) from the European Union (EU), has impacted socio-political relationships, both internally, and externally with other countries and economic groups. This has been especially true regarding international trade, and legal and market standards for food and food security. This paper examines how the enacting of Brexit has framed and underlined contemporary perceptions of the UK neoliberal food system, the relative importance of food standards, and the impact of policy transition on food security. Using a positional approach, perspectives and narratives within the literature are critiqued and synthesized, including academic sources, parliamentary debates, economic reports, and media analysis. The politico-economic effects of Brexit have altered food-related relationships, recalibrating trade interactions and changing the public funding that UK farmers receive. Through realigning extractive economic models, the pre-Brexit UK food system has been reset, and new perspectives about neoliberalism have emerged. Government intervention has steered away from traditional neoliberal framings towards neo-developmentalism. A dichotomy thus exists between recognizing the intrinsic right to adequate and nutritious food and maintaining existing cultural dynamics of food supply, and the use of agri-food policy as a politico-economic tool to drive higher economic growth. The implications of this policy change are stark for UK agri-food actors within food system transition post-Brexit.

## 1. Introduction

The stability of the UK food supply involves two key elements: the safety of supply and the security of supply. Food safety constitutes a foundational element of food security. Food safety refers to the production, manufacturing, processing, packing, or holding practices recognized as leading to the provision of safe (and wholesome) foods. The consumption of ‘safe foods’ under normal circumstances and in normal quantities should not lead to health problems for the consumer [1]. Setting standards regarding the safety and legality of food, compliance with food hygiene rules, and the provision of accurate food information to consumers are critical to the safety of food [2] and these issues are in principle governed by food law. The United Nations Food and Agriculture Organization (FAO) definition of food security is as follows:

“Food security exists when all people, at all times, have physical and economic access to sufficient, safe and nutritious food that meets their dietary needs and food preferences for an active and healthy life.”

[3]

Compliance with food standards and the stability of food supply in the UK have had tumultuous histories. Microbiological and contaminant-related and animal disease-related food scares have been witnessed, as well as weak food quality standards and animal welfare issues that have affected the stability of the food system [4]. In the 1980s, *Salmonella* and *Listeria* outbreaks became safety concerns that received significant media attention, and the ‘egg scandal’ caused the then Health Minister to resign [5,6]. In the 1990s, concerns arose over bovine spongiform encephalopathy (BSE) and its impact on public health, and *E-coli* infections were recorded in Scotland at the second highest reported rate of infection globally [5,7]. The broad geographic spread of animal product-related food incidents led to compulsory animal identification systems being introduced in the UK and wider European Union (EU) to allow the full traceability of animals and animal foods, which increased confidence in trade [8]. EU food law rapidly evolved from being market-oriented to being food safety-oriented; in the UK for example, these concerns led to the introduction of the Food Safety Act 1990. Indeed, since the 1990s, official controls for food of animal origin in Europe have been much more detailed and demanding than for products of plant origin [9].

Throughout the early 2000s, ongoing food safety incidents continued to arise in Europe and other parts of the world [10]. In June 2013, facing the European Horsemeat Scandal, the UK Government commissioned a report on the integrity and assurance of food supply networks [11]. Despite the UK’s food supply system being described in this and subsequent publications as ‘one of the safest… in the world’ and ‘highly effective and resilient’ [11,12], recent years have witnessed not only the major disruptors of the 2007–2008 financial crisis, Brexit, the COVID-19 pandemic, and the Ukraine/Russia conflict, but also rising dependence on food banks, increasing reliance on free school meals, acknowledgement of the vast consumption of processed food in the national diet, and a contested discourse around post-Brexit trade deals with the potential to lower food standards further [13,14], all potentially threatening individual/household/national food and nutritional security.

Brexit was the name given by the media to the UK’s withdrawal from the EU, which took place on 31 January 2020. This followed the 2016 national referendum on membership of the EU, where 51.9% of those who participated voted to leave the EU, and 48.1% voted to remain [15]. The referendum results varied considerably across regions of the UK [16], i.e., Scotland and Northern Ireland both demonstrated majority support for remaining in the EU; however, these regions have relatively small populations and thus a smaller impact on the final result as England has a significantly larger population [15]. Politico-economic instability negatively affected the cost of living and real-value of wages in the UK prior to the withdrawal [17]. The leave vote led to a substantial depreciation of the value of sterling, which had an upward effect on consumer prices, especially in product groups with a higher import share (such as clothing and footwear, and food and drink). The concurrent geopolitical–economic influences of Brexit and COVID-19 make it difficult to determine the distinct impact of Brexit from amongst other drivers. Since the COVID-19 pandemic, when food shortages became a reality in certain areas of the UK, there have been calls to enshrine the right to food into national law [14,18]. However, there is still a dichotomy between the deontological basis of food provision and the politico-economic situation causing food provision to become one of the most contentious weapons within global politics [19]. The use of food sovereignty and rights of access as a political tool is an age-old strategy, as convincingly demonstrated during the Brexit negotiations, especially regarding the positioning of fishing rights in UK waters, a topic fueled by UK–Jersey–France tensions [20,21]. Food sovereignty, i.e., “the right of people to healthy and culturally appropriate food produced through ecologically sound and sustainable methods, and their right to define their own food and agriculture systems” [22], (p. 1), has a historical basis. However, as a topic of interest, it has been “back in vogue” in the face of the dominance of transnational corporations in food value chains and the challenge of climate neutrality. Brexit previously divided, and still divides, opinion among the public, academics, politicians and food producers [23], and the use of food as a politico-economic bartering tool is a concern for UK trade deals and the impact on consumer health [24], and food choice. As Millstone et al. [25] (pp. 645 and 653) wrote,

“Brexit’s impact on the UK food system is immense because food has been highly integrated into EU governance […]” and the major considerations include food standards and food security, which have high public salience, as well as others that are less widely debated, such as the future of agricultural subsidies.”

The aim of this position paper is to reflect on how the enacting of Brexit has framed contemporary perceptions of the UK neoliberal food system, the relative importance of food standards, and the impact of the policy transition on food security, of which food safety and quality are sub-components. A position paper is a type of academic writing that supports the authors’ position on a topic by presenting well-researched, relevant evidence. A position paper is a form of communication where authors take an active part in dialogue with a scholarly community and take a stance on a chosen topic and defend their position with qualitative and quantitative research from scholarly or academic peer-reviewed sources [26]. Considering the impact of Brexit on UK food standards and food security through the lens of neoliberalist policies and practices reveals how ideas of neoliberalism and capitalism can be conflated to imply they are symbiotic, but here we position that neoliberalism is a political construct and that capitalism is an economic system. Thus, capitalism as an economic system is influenced by, and itself influences, neoliberal practices, or even what is considered as being neoliberal. How neoliberalism frames the operationalization of the UK agri-food system involves the contestation between a neoliberal food system and food justice [27].

Recent crisis situations have not only overlapped and even mutually intensified each factor, but they have also exhibited some common features, i.e., operators in food supply chains have faced serious disruptions arising from shortages of materials, resources, and workforce, etc. Brexit and the COVID-19 pandemic caused the UK Government to encourage British consumers to ‘buy British and local’, but the pandemic then made that difficult when salaries we reduced during furlough, mirroring what happened after the 2007–2008 financial crisis. However, Duncan [28] differentiates between the narrow neoliberal ideology applied in 2007–2008, with associated ‘austerity policies’, and the initial responses of the UK Government to the COVID-19 pandemic, with a form of neoliberal governmentalism framing their approaches post-pandemic. Whether future UK food policy presents the death of neoliberalism with the rise of interventionism or a reimagining of contemporary neoliberalism is an interesting question. Möller [29] (p. 864) describes it as a movement from a reluctance for direct government intervention to being “in favor of more distanced manipulations and constructions of market environments built upon the principle of not governing too much”. It is certain that this ‘perfect storm’ still presents a unique and complex example of the challenges associated with the UK agri-food system, food standards, and food security in the UK, one that is worthy of study and academic reflection. However, it is difficult to determine the discrete impact of Brexit given other politico-economic shocks and squeezes. This paper makes a contribution by considering emergent descriptions of neoliberalism such as eco-extractivism, eco-imperialism, and neo-developmentalism, which are explored in the context of evolving UK food policy, especially the risk of developing a ‘two-tier’ food system. A series of novel research questions emerge from this position and are considered in this research:

1. How are the politico-economic relationships around food affected by the implementation of Brexit, and then compounded by other recent shocks?
2. Why are the paradigms influencing contemporary politico-economics framed around food price rather than national food security?
3. What forms of neoliberalism are emerging in the post-Brexit UK agri-food system?

The paper is structured as follows: Section 1 is the introduction and Section 2 outlines the methodological approach. Section 3 outlines the theoretical framing, and Section 4 concentrates on a UK perspective. Section 5 is the discussion. Concluding thoughts are presented in Section 6.

## 2. Methodological Approach

This position paper was developed to advance a structured narrative and a series of arguments on the issue of the influence of Brexit on UK food standards and the access to safe and sufficient food in the light of changing neoliberal framings. Secondary data described in the gray literature and other evidenced sources are synthesized via an iterative approach to frame the three emergent research questions and then address them [30]. The key search terms were Brexit, food security, food standards, neoliberalism/neoliberal/neoliberalist, and neo-developmentalism. The following databases were searched: Science Direct, Google Scholar, Google (including the gray literature). The keywords were used in a range of combinations of the search terms, i.e., through an iterative literature review method. The first fifty items in each search were considered for relevancy and any duplication. All pertinent papers were subsequently compiled, and their titles and abstracts were read. The full texts of potentially relevant papers were then examined to assess their relevance and contribution to the development of a discursive narrative and argument pertaining to the issue addressed in this study. As a consequence, 112 scientific papers and gray literature publications from government, academic, business, and industry sources were used to support this paper. The literature review, on the basis of which the initial version of the text was prepared, was conducted in the last quarter of 2024. The first draft has been considerably improved thanks to the literature searches that were conducted in the first quarter of 2025. The quantitative data were derived from internationally recognized sources such as the UK Department for Environment, Food & Rural Affairs (Defra), the UK Department for International Trade, the UK Parliament, the UK Office for National Statistics, FAOSTAT, and the Economist Impact.

## 3. Theoretical Framing

### 3.1. Neoliberalism and Capitalism

Neoliberalism may be defined as follows:

“A set of political beliefs which most prominently and prototypically include the conviction that the only legitimate purpose of the state is to safeguard individual liberty, understood as a sort of mercantile liberty for individuals and corporations… that the state ought to be minimal or at least drastically reduced in strength and size, and that any transgression by the state beyond its sole legitimate raison d’être is unacceptable.”

[31] (p. 203)

Neoliberalism has dominated economic political policy in the UK since the era of Thatcher and Reagan, but it encompasses multiple economic perspectives such as Keynesian economics and regulatory governance, incorporating policies like quantitative easing (QE), the lockdown response to COVID-19, and the furlough scheme. The contemporary key national and international features of neoliberalism are synthesized in Table 1.

Yet, given these disruptive geopolitical trends, state intervention in the food economy is inevitable almost everywhere in the world through either direct payment to farmers or other public support or taxation mechanisms. However, this approach is incompatible with neoliberal policies, which seek to minimize state intervention. Some would argue that whilst neoliberal policy supports the workings of free-market capitalism, it also supports the regulation of national and international trade to safeguard the rights of individuals, champions individual liberty and well-being, and achieves a more efficient allocation of resources [31,32]. Therefore, should neoliberalism support capitalism when it infringes on these rights? Recent reflections on capitalism suggest that multiple elements have the potential to infringe on individual’ rights: (1) the pursuit of profit and its private appropriation, (2) free enterprise and the competitive market, (3) the payment of wages for labor and the production of commodities, (4) property rights, (5) the financial infrastructure of money and investment that enables credit and debt, (6) a highly variable degree of state regulation, and (7) a propensity for growth as the productive re-investment of profit [33].

Whilst some hail the ‘seeds of a potentially post-capitalist [urban] society’ [34] and aim to remove from the food system the ‘vestiges of capitalism’ [35], others demonstrate that, due to the narrow understanding of the concept of neoliberalism, its full potential has not been harnessed, and potential opportunities have been ‘overlooked’. Indeed, Adam Smith, the first theorist of capitalism, believed in basic wages that were high enough to support the necessities of a decent life, food being one of these, and the idea of a moral economy being rooted in capitalism. Porter and Kramer [36] (p. 324) explained how capitalism itself is not the ‘enemy’ but instead is an ‘unparalleled vehicle for meeting human needs, improving efficiency, creating jobs and building wealth’. Thus, this paper positions that whilst neoliberalism and capitalism are cited by some as being the reason for the inequalities and inequities of the food system, it is prudent to remember that it may be intentional or unintentional socio-economic distortion of the concepts rather than the construction of the ideologies themselves which has led to these outcomes.

At the time, the UK Government was able to portray Brexit as an unprecedented opportunity to address the ecological disbenefits of agricultural productivism (including, by implication, climate change) since, according to neoclassical and neoliberal economic doctrine, these were the outcome not of capitalism but rather of the continuing market interventionism (statism) of the EU’s common agricultural policy (CAP), i.e., element 6 of the aforementioned definition of capitalism from [33]. This rhetoric suggested that, by removing such interventions, the “free play’ of market forces would then secure that axiom of neoclassical theory: ‘optimal allocation of scarce resources’” [37] (p. 6). Within this rhetorical approach, any environmental ‘market failures’ could then be made good with state subvention for ‘public goods’. Tilzey [37] argues here that there is a developing perception of the concept of neoliberalism based on aspects such as political productivism (e.g., land intensification for food production, and land sparing for nature), rather than more holistic land sharing, and on market productivism, reducing the intrinsic environmental footprint of products. This new form of radical neoliberalism, especially with regard to rural policy and the influencing role of special interest groups, becomes a quasi-hegemonic environmental neoliberalism, with the phasing out of former direct support payments to farmers within England, originally set for 2028. In October 2024, in England, the delinking and phasing out of direct support payments was accelerated. The associated land use changes this policy agenda affords frees land within England for other infrastructural and policy-driven uses including reducing national greenhouse gas emissions and supporting the delivery of national (UK) net zero and biodiversity recovery targets. However, some argue that this leads to the expansion of environmental imperialism (eco-imperialism) by offshoring the negative externalities of food production associated with food imports [38]. This highlights a localized form of neoliberalism with a deconstruction of local barriers to economic growth and global neoliberalism approaches where technofixes operate within countries, and across borders, to deliver specific outcomes including food security [37,39].

### 3.2. Human Rights Approach to Food Security

In the era of turmoil and crisis in the financial markets resulting from public health events, political issues, extreme weather events, pest infestations etc., causing disruption to supply chain function, some suggest maintaining a high degree of national food self-sufficiency is key to ensuring food and nutrition security. Doherty et al. [40] use the 2007–2008 financial crisis to highlight how the food price spike led to the UK households buying less food overall (4.2%), and also buying cheaper alternatives, with the poorest 10% of people seeing their relative food bill increase by 40% more than the average between 2007 and 2011. The associated calorie and nutritional insecurity indirectly led to public health issues, exacerbating the divide between those who can, and cannot, afford to be food secure.

Lockdowns (school closings, travel bans, quarantines, etc.) associated with COVID-19, growing energy and food prices, fertilizer price inflation, and other problems that occurred in relation to the latest pandemic, Brexit, and war in Europe have influenced all the dimensions of food security, i.e., availability (based on supply of food which is adequate), access (people should obtain the food they need), utilization (the proper intake of nutrients), and stability (people should be able to access food at all times) [41,42]. The UK Global Food Security Index [43] was 78.8 in 2022, which positioned the country in 9th place out of 113 countries. The UK performed the strongest in the Affordability pillar (91.5/100) and weaker in the other three pillars, i.e., Quality and Safety (80.1), Availability (71.6), and Sustainability and Adaptation (71.1). The UK’s high affordability score was due to very good performance in metrics such as average food cost, food safety net programs, and the proportion of the population under the global poverty line. The Quality and Safety sub-index in the UK was lower than the European average. Micronutrient availability (vitamin A, iron and zinc) and dietary diversification (share of non-starchy food and share of sugar consumption) significantly lowered the UK’s national score [43]. This nutrient insecurity score aligns with 38% of UK adult citizens being overweight and 29.5% of adult citizens being obese in 2021, and 37.7% of children being overweight or obese, as reported by the Nuffield Trust, an independent health think tank in the UK [44].

According to data supplied by FAOSTAT [45], the share of obese people in the adult population in the UK (18 years and older) consistently grew year on year, from 19.1% in 2000 to 26.8% in 2022. In this regard, in 2022, the UK Government released a plan on the non-promotion of foodstuffs high in fat, salt, or sugar by restricting volume offers like ‘buy one get one free’. However, the implementation of this plan was delayed until October 2025. The Global Food Security Index [43] for the UK was higher in 2022 than the 2017 value, which was mainly due to the increase in the Sustainability and Adaptation sub-index. Major improvements were made in the area of disaster risk management and political commitment to adaptation. Further, the UK Availability score was higher in 2022 than it was in 2012 due to a high agricultural R&D sub-index, but it also is attributed to better supply chain infrastructure, better access to agricultural inputs, lower political and social barriers to accessing adequate food, and lower food loss. The high volatility of agricultural production appeared to be the most pressing problem in terms of ensuring food availability in the UK.

Food forms the basis of survival and yet, arguably, is still treated as an auxiliary luxury from an economic theory perspective. As a result of the great harm that human beings experienced during World War II as a result of food shortages among other factors, the 1948 Universal Declaration of Human Rights (UDHR) in Article 25 enshrined the right to food:

“Everyone has the right to a standard of living adequate for the health and well-being of himself and of his family, including food, clothing, housing and medical care and necessary social services, and the right to security in the event of unemployment, sickness, disability, widowhood, old age or other lack of livelihood in circumstances beyond his control. Motherhood and childhood are entitled to special care and assistance. All children, whether born in or out of wedlock, shall enjoy the same social protection.”

[46]

It is worth noting that the UDHR is not legally binding, but it does set out the basic norms and standards to which all countries are expected to adhere. It has been followed and supported by other UN legislation, including several international legal instruments that are legally binding, in particular, the 1966 International Covenant on Economic, Social and Cultural Rights (ICESCR), and the 1989 Convention on the Rights of the Child. Although a right to food is not featured within the European Convention of Human Rights, nor within the UK Human Rights Act 1998, internationally, a “right to food” exists within article 11(1) of ICESCR, which that states a “right of everyone to [an] adequate standard of living… including adequate food…”. Article 11(2) adds that states must recognize the “fundamental right of everyone to be free from hunger” and take measures to improve methods of food production, conservation, and distribution [46].

The right to food requires countries to provide an effective policy and regulatory framework, intervene in situations of market failure, and create an enabling environment in which food security is ensured. It is any state’s obligation to help to overcome impediments in any of four food security dimensions, i.e., availability, accessibility, utilization, and stability.

### 3.3. Trade-Based Drivers of Food Security

When considering UK food consumption, over 2/3 of the UK’s land footprint is outside its national boundaries [47], creating positive and negative externalities across the world, as well as driving narratives focused on offshoring and eco-imperialism. Soya and rapeseed accounted for the largest increase in greenhouse emissions associated with UK agri-food supply from 1987 to 2008, being amongst the top 10 imported crops, which are collectively responsible for 3/4 of the UK’s total cropland footprint overseas [47]. Food trade in this context is a tool to drive economic growth and profit, allowing corporations directly, or indirectly, to control food access, health, and wages, but there is growing pressure too for businesses to demonstrate that Environmental and Social Governance (ESG) strategies are in place. This debate is heated, with questions around trade-offs between sustainable long-term food security solutions versus capitalist short-term gains. Pressure is placed on supermarkets, private companies, and governments by the public in a discourse that often fails to highlight the complexity of the challenges the market is being required to meet [14].

Even though the UK is around 75% self-sufficient in foodstuffs that can be produced domestically, the UK is only 17% self-sufficient in fresh fruit [48]. A comparison between 2021 and 2023 (Table 2) shows a similar trend between years for some commodities, but for others, such as pork, poultry (meat and eggs), and vegetable production, a downward trend is visible [49]. When considering fruit and vegetables, the percentage of production to supply met by produce from the EU was 39% for fresh vegetables and 27% for fruit [50]. In terms of overall food consumed in the UK, 58% was from the UK, 24% was from the EU (24%), and then supply was from the rest of the world [50].

The specific geographical constraints of the UK as an island, the reliance of the UK’s agri-food sector on a foreign labor force, high input prices (incl. land, labor, fertilizers, pesticides and fuel), and a focus on reducing national greenhouse gas emissions to deliver net zero have been major obstacles to increasing self-sufficiency in certain foodstuffs. However, according to the Cabinet Office, the ‘geographic diversity’ of food sourcing contributed to the UK’s ‘highly effective and resilient food supply chain’ [12]. Pre-Brexit, with just under 1/3 of food consumed in the UK originating in the EU, another 11% came from trade deals negotiated by the EU with other countries [51]; when added to imports of animal feed, this suggests a UK international food-related dependency of around 50% [47]. The value of the UK’s food imports represented almost 9% of the total value of imported goods in 2023, and vegetables and fruit (top trading partners: Spain and Netherlands) and meat products held the lion’s share of the food products imported (top trading partners—Irish Republic, Netherlands, and Poland) [52]. The UK’s import of food and live animals from the EU, in terms of value, was more than twice the value of such import from non-EU countries over the period 2022–2024, which was due in part to the high and fluctuating costs of goods transport. Brexit also initiated a change in the list of major food suppliers to the UK market, e.g., the provision of agri-food supplies from Poland to the British market increased [53]. Furthermore, the UK started to build new and innovative trading relationships with countries outside the EU, drawing on its internationalist heritage [54]. The development of the UK’s domestic agri-food policy post-Brexit and post-COVID-19 is now considered in more detail.

## 4. The UK Perspective

### 4.1. Agricultural Policy Reform

Politically, the agricultural policy for the four nations within the UK is diversified. The 2020 Agriculture Act, S. 1, encourages farmers in England to deliver to market standards, but also to deliver in terms of the use of environmental measures. This regulatory approach provides an opportunity for both neoliberalism and capitalism to be repositioned by an alternative politico-economic paradigm that captures environmental and social value via the notion of ‘public funds for public goods’. The Agricultural Act’s provision proposes that the Secretary of State should report to Parliament on food security at least every 3 years (S. 19(1)), although this frequency has now been increased to annually [55,56]. The first index was published in July 2024 [55]. Additionally, enabling the support of innovators and entrepreneurs within S. 1(2) may demonstrate that food production is crucial, not only in post-COVID-19 and post-Brexit economic recovery, but also for long-term sustainable national food provision. However, it is not clear whether the terms laid out under S. 1(2)(b) address funding opportunities for farm businesses to maximize the value of the raw ingredients they produce. For instance, S. 20 states that intervention in agricultural markets must only be in exceptional market conditions (such as the COVID-19 pandemic), and not in response to weather or animal disease per se, unless they result directly in market disturbance. However, the legislation stops short of safeguarding the intrinsic value of food, with the first version of the Food Security Index in 2024 being based on nine indicators relating, primarily to economic and supply-based (availability) metrics rather than calorie or nutrition security at the individual or household level, i.e., food provision is not explicitly stated as being a public good. Combating hunger and malnutrition is a legally binding human rights obligation in the UK and many other countries [46], and the United Nations Sustainable Development Goal (SDG) Number 2 is focused on creating a world free of hunger. It can be argued that food and nutrition security should be treated as a public good [42] or a global public good [21]. More public and regional developments have emerged, however, with Sustain’s Right to Food Campaign and Manchester City Council supporting the campaign to end food poverty, pressuring the Government of the time to enshrine a right to food within UK legislation, with Manchester itself becoming a ‘right to food’ city [18,57].

Ensuring the maintenance of rigorous food-related safety and quality standards is a topic of much debate in the UK. Indeed, the Agriculture Act 2020 stipulates under S. 42, that the Secretary of State must report to Parliament on whether, or to what extent, provisions in free trade agreements (FTAs) relating to agricultural products are consistent with the maintenance of the UK statutory levels of protection in relation to human, animal, and plant life or health, animal welfare, and the environment. However, it does not categorically state that the UK cannot import from countries with lower food quality standards. This could provide a legislative loophole that undermines existing national food standards and demonstrates the potential for the politicization of food commodities in negotiations where agriculture and food supplies are bartered in a trade agreement for UK access to other markets, e.g., finance or services sectors. Arguably, therefore, this creates vulnerability. both for UK food producers and for consumers who may be unaware of the standards of food being imported. Food safety and the protection of public health are worldwide priorities; however, when food is scarce or consumers are facing the problem of high food inflation rates and/or food shortages, there are downward pressures on price, which can impact food standards, especially the animal welfare and nutritional standards of the products available for purchase.

The possibilities of the UK home market being undercut by more cheaply produced imported food could negatively impact on the UK farming sector if it aligns with a failure to develop export markets for foods produced to higher standards in the countries with which FTAs are being forged. The implementation of higher UK farm standards within a market that does not financially reward them and exerts pressure to make space for other land-based services, e.g., carbon capture, renewable energy, or biodiversity restoration [58], and increased demand for housing as the UK population increases, will drive change in land use demands and potentially the provision of food for domestic or export markets. Similar trends are being witnessed in Europe, with the political desire for environmental protection usurping the desire for food production, with one view being that policy is “turning proud and independent food-producing farmers into publicly paid ‘landscape gardeners’” [59] (p. 37). The potential for such dichotomic changes to threaten rural livelihoods, mental health (and feelings of identity), and food system resilience is high, as is the risk to the rural economy. As Henry Dimbleby states in the UK National Food Strategy, “farms are businesses, not philanthropic hobbies” and we cannot expect them to become sustainable if it “destroys their balance sheet” [58] (p. 11). These examples highlight the emergent reframing of the UK neoliberal food supply system and the likely trade-offs around the introduction or reintroduction of wider land use policy, such as in England with the Land Use Framework consultation [60], and potential technological solutions that address food security, such as gene editing and cultured meat production, as collective societal discussions are mediated by complex and contradictory worldviews [39].

The Government’s approval of, or at the least acceptance of, lower-quality food imports and the standards-based equivalence of international competitors is impacting UK farmers and producers. At a time when Government area-related farm support in the UK, as a legacy of being a member of the EU, is reducing or ceasing, depending on the policy in a particular home nation, the support of family farming businesses through historic tax breaks is also being severely constrained. Considered alongside the rise of competing demands for land use, this is a difficult time for farming businesses. The Defra data for livestock numbers in the UK for June 2024, prior to the Autumn budget tax changes, highlighted the decline in livestock numbers in the UK.

Pig numbers increased in England by 1.3% between 2023 and 2024 (but breeding pig numbers fell by 1.0%).

Cattle and calves’ numbers in England decreased by 2.0% to 5.0 million between 2023 and 2024 (the breeding herd decreased by 2.2% and the dairy herd was broadly stable);

Sheep and lamb numbers in England decreased by 4.3% between 2023 and 2024 (breeding flock (females) dropped by 5.6%):

Broiler (meat) chicken numbers fell by 3.5% between 2023 and 2024. This was before the market-driven depopulation of poultry meat housing in early 2025 (from 38 to 30 kg/m^2^). Laying hen flocks remained stable, with turkey numbers increasing by 25%, although this represents about 2% of total poultry on the ground [61].

Whilst the reduction in livestock numbers in England may be welcomed as an intervention to deliver national net-zero greenhouse gas emission commitments by 2040, and contributing to meeting the Global Methane Pledge in 203, and the ‘30 by 30’ (or 30 × 30) biodiversity initiative in 2030, total meat production in England fell by 3.7% in 2023 [62]. This downward trend in UK-produced meat is important to follow going forward (see Table 2), as are the changes in the total value of the market due the reduced supply, the mechanisms that use imports to substitute in the food market, and their impact on affordability and availability. Changes in the patterns of production and consumption of meat in the UK, with a significant increase in poultry consumption and a substantial decrease in red meat consumption, could be the way to meet the dual goals of food security and net zero [63]. The current UK Government has sought to stabilize concerns over the financial viability of UK agriculture [64]. However, the neoliberal approach to support mechanisms for UK agriculture, as well as the changing regulatory environment around land use and the delivery of nature-based targets, is creating uncertainty in an industrial sector that needs continual investment for the long term. The form of food neoliberalism described herein is different to a process of regulatory simplification and reducing government intervention to free up food supply and trade. This neoliberal intervention is amidst a mosaic floor of agri-food and land use policy in the UK, consisting of both eco-neoliberalism through both direct intervention from the market and also the indirect intervention from citizen scientists, non-governmental organizations, and supranational organizations. Indeed, as they seek to increase their political agency, demonstrations by farmer representative groups around the world in 2024 and 2025 are aligned with counternarratives of food democracy, food sovereignty, and land sovereignty [65]. Omar and Thorsøe [66] (p. 631) conclude the following when considering the EU farm-to-fork strategy:

“Our analysis found that the discourses, namely “rebalance power in food system” and “strengthening farmers’ position in value chains”, are marginalized in favor of an innovation-investment discourse, indicative of greater financialization and technologization based on techno-finances fixes in transforming the European Union agri-food system. We argue that entities representing agri-business interests have been influential in the policymaking process and voices representing smallholder and medium-sized farmers’ transformational discourses have been excluded.”

The range and volume of voices and modes of expression in the discourse on agri-food transition, and how often farmers’ voices are absent, have also been considered by others [39]. Mass protests of farmers in many EU countries caused the required transfer of arable land to non-productive purposes related to eco-schemes to be temporarily removed in 2024 [67,68,69]. The question arises herein as to whether these complex and competing demands for food and land use lead to more, or less, resilient national, regional, and global food systems. The next section explores the changes in the interaction between the UK and the EU post-Brexit and the implications for food security, with a focus on resilience.

### 4.2. Agri-Food Trade Relations Between the UK and EU

Since becoming an independent trading nation, the UK could have either followed the predominant ‘free-trade’ neoliberalist trajectory or create a different politico-economic neoliberal path towards a more self-sustaining, resilient food economy. In 2019, Chatham House highlighted the difficulties that Brexit posed for trade and requisite targets, including maintaining existing and future standards with the rest of the world, negotiating a better EU27 trade deal, and promoting frictionless trade, primarily with the EU, via regulatory harmonization [70]. Despite the UK no longer being part of the EU customs union or single market, though the EU–UK Trade and Cooperation Agreement (TCA), operational from 1 January 2021, ‘100% tariff liberalization’ was agreed, i.e., there would be no tariffs or quotas on the movement of goods across borders, easing fears of large tariffs being imposed post-transition [70,71]. The European Commission [72] highlighted that under World Trade Organization (WTO) rules, without the TCA, ‘beef, dairy, poultry, pork, lamb, cereals, sugar and several processed foodstuffs could have faced tariffs of some 50% or above’. Nevertheless, despite this agreement, there are additional customs controls and new procedures for moving goods, including food, between the UK and the EU, creating further costs and bureaucracy [73]. The introduction of the UK technical regulations and regulatory compliance checks for incoming EU *goods* and live animals and low-risk plant checks were delayed until 2022 [74]. Whilst such delays aided the delivery of UK food security by reducing friction at borders, the impact on UK food system resilience was more concerning. For example, whilst EU pork imports to the UK did not require checks, UK pork exports to the EU needed to comply with more expensive and burdensome regulations [75], so UK farmers were continually outcompeted. This supply and demand problem was reinforced by the ease for EU exporters to replace more demanding UK buyers with alternative recipient countries within the EU, whilst UK exporters struggled to find “local” replacement markets [76]. It could be argued however that the adoption of additional regulatory controls (veterinary attestation visits) in the UK, administered by veterinarians on livestock farms, may actually have a long-term positive benefit in terms of animal welfare, animal health, and improved productivity gains [77]. Furthermore, after Brexit, for goods crossing the UK-France/Belgium border, each country generalized data sharing and pre-lodgement through “smart bordering” models and information technology programs, e.g., the Goods Vehicle Movement Service (GVMS) system [78]. The programs were implemented and are still being upgraded under a smart bordering strategy. For private and public actors, including producers, transporters, and customs, avoiding physical controls at ports and further improving mutual cooperation became an imperative after Brexit. However, the approach to the interaction between the UK and EU in terms of agri-food policy is still open to reconfiguration, but being smarter in terms of data sharing and regulator systems is essential.

### 4.3. Post-Brexit Global Trade Deals

The COVID-19 pandemic highlighted and accentuated issues created by Brexit relating to UK food security/insecurity [23,79]. There was contestation between the provision of cheaper, lower-quality food on retail shelves for a proportion of the population and the high-quality food agenda, supporting home-produced food and food exports from the UK. Indeed, UK policies to promote healthy eating may be considered barriers to trade by potential trading partners [23]. There was also a dispute when the EU–US negotiations were taking place for the stalled T-TIP or Transatlantic Trade and Investment Partnership over whether Investor–State Dispute Settlement (I-SDS) mechanisms would form part of the trade deal [80]. Thus, I-SDS disputes could potentially reframe existing interactions between sovereign governments and corporations.

Negotiations on trade deals between the UK and New Zealand (NZ) intensified from 2020. A NZ–UK trade agreement entered into force on 31 May 2023. Since then, both countries have had preferential access to a consumer market in a second part of the agreement, which could be seen as a British gateway to Asia and the Pacific and a gateway for NZ into the UK consumer market [81,82]. However, this trade deal had many dissenters, especially UK food producers, due to market access differences between them and their NZ counterparts, particularly in beef and lamb production [83,84]. With regard to the UK–US food trade deal, narratives around the impact on UK food standards centered around ‘chlorinated chicken’, with 85% of respondents in a 2019–2020 study stating it should be illegal to sell this product in the UK [85]. Any UK–US bilateral agreement is framed by these complex narratives about food standards, relating to food safety, public health, and animal welfare, and such agreements, some suggest, need to consider the views of citizens [70], or there is a risk of growing public distrust of the food system and ultimately, the Government. At the time of writing, the US has introduced a range of trade tariffs, including for the UK, and their impact on food security and potential future trade deals is yet to be quantified.

The UK–Japan trade deal and the UK–Australia deal have also been formalized. However, there were concerns regarding the omission of climate change targets in the UK–Australia deal [86], as Australian beef has a higher carbon footprint than UK beef, partly due to accounting for deforestation to create pasture; thus trade could disrupt the UK national food-related environmental balance sheet [58] or give rise to concerns over exporting the carbon footprint. Despite this, the then Government said it was committed to protecting UK food standards. However, Luke Pollard stated that ‘it is clear that despite having one Government… [there are] two competing food agendas’ [87], namely producing high standards of foods for export whilst accepting lower standards of foods for consumption at home. In 2018, 9% of UK agriculture and food exporting businesses exported to Japan, with 2% of importing businesses in the same sector were also importing from Japan [88]. Whilst the ability to agree such trade deals swiftly is ostensibly beneficial, a politically pressurized ‘hasty decision’ could lead to poorly constructed policy that is then difficult to adapt [70]. However, whilst this deal may have minimal benefit for UK food security and food system resilience, the focus on exports of food from the UK to Japan is of interest given the low level of Japanese food self-sufficiency. Thus, UK food exporters may benefit by reducing tariffs on products like beef and salmon and boosting the Japanese market for high-value products like whisky [89]; the reverse benefits for UK consumers are limited given reduced tariffs for products such as udon noodles and fresh Pacific bluefin tuna.

### 4.4. Neoliberalism, Neo-Developmentalism and Neo-Extractivism in the Content of Food Systems

Whilst not directly aiding or benefiting UK food system resilience, the development of trade deals and their use as a political tool may prove advantageous to long-term UK politico-economic resilience. Herein, the focus on politico-economic resilience is different to the positioning of the state or the equilibrium of ‘resilience’ in the context of a food system, i.e., “the capacity over time… to provide sufficient, appropriate and accessible food to all, in the face of various and even unforeseen disturbances” [90]. We contend here that politico-economic resilience is the state or equilibrium that results from combined political and economic actions that together promote national or regional resilience in terms of being able to prepare for, withstand, respond, and adapt to, and recover from severe and/or prolonged geopolitical and socio-economic squeezes or shocks. The position here is that the food system does not operate in isolation from other systems such as the natural system, financial system, and/or the political system [91]. Thus, national neoliberalism (articulated by different political parties within a given country) interacts with global neoliberalism (promoted, articulated and deployed by an architecture of transnational and supranational organizations), and with one aspect in particular, neo-developmentalism [92]. Neo-developmentalism, within economic policy, is focused on state ‘intervention’ via political and industrial strategies aimed at ensuring “credibility with financial markets, trade and financial openness, internal and external competitiveness” [92] (p. 98). Ban et al. [92] argue that neo-developmentalism, as a form of neoliberalism, is focused on a rebalancing of labor and capital to drive competitiveness and the realignment of tax regimes from the supply side to taxation on capital and assets. This extends notions of contemporary neoliberalism from the binary aspects of neoliberalism or neo-statism to considering neoliberalism as a mechanism for the politico-economic reconstruction of power relations [93].

Neo-developmentalism, a theory driven by Latin American countries, strengthens the role of the government in the market, can narrow the gap between the rich and the poor, and improve the quality of life for people within currently disadvantaged communities, but can make national economies more vulnerable to global markets and external shocks [94,95]. Neo-developmentalism has also been linked in the food system context to aspects of neo-extractivism, specifically agro-extractivism through a specific policy of exporting food products to generate economic value within a given country, especially in Latin America [96]. Thus, progressive forms of neo-extractivism, i.e., the marketisation of natural capital via food and carbon markets, allows “extractivist production to continue to thrive while [the government is] using the … income it generates to fund social programs” [96], (p. 426). Neo-extractivism is at odds with food sovereignty and wider calls for national food self-sufficiency [97]. In the UK, self-sufficiency as a food system resilience strategy is not a favored policy [58,70]; instead, food supply is met by policies of equivalence, not complementarity, reducing food standards via food imports, meaning that a two-tier food system is created. Household food insecurity in the UK has risen over this time frame. In 2020, 2.9% of adults in the UK in one survey stated they had not eaten for a whole day; by June 2024, this had risen to 4.8%, and households with children were more likely to be food insecure than those households without [98]. Overall, compared with the 7.8% rate in January 2022, 12.2% of households were having smaller meals or skipping meals in June 2024, peaking in September 2022 at 17.6% [98]. The number of children eligible for free school meals increased from 17.3% in 2019/2020 to 24.6% in 2023/2024 [99]. Comparing 2020 to 2023, the volume of food purchases in the UK for certain foods fell: fish (15.1%), milk (12.2%), fruit (12.1%), and bread (11.3%). Others saw changes of 10.7% and below. Confectionary and pastries saw the lowest drop in volume (around 1%) purchased [99].

Agro-extractivism has been linked with large-scale corporate monoculture agriculture, where the business model is one of extractive capital accumulation [100]. Indeed, narratives around regenerative rather than extractive food systems (productivism) have gained ground in the last decade [101,102]. There are growing numbers of legislative bodies, civil society organizations, and consumer groups supporting ‘regeneration’ to deliver a resurgence of fortunes for UK producers, nature and food production. These actors support environmental protection and high UK food standards, aiming to improve the resilience of the national food supply [25,58,79,103,104,105]. A program of UK food system ‘decentralization and diversity’ has been suggested to reduce the reliance on large retailers, with the particular aim of delivering food security [14]. Indeed, the UK Agriculture Act 2020 recognized the importance of diversity within a resilient food system, with S. 1(2) aimed at increasing the diversity of producers and the numbers of smaller enterprises [106]. In this politico-economic environment, uncertainty has remained the prevailing feeling for many agricultural businesses [14,79], especially considering the varying trade deals, changes in the tax system, insecurity in terms of agricultural worker numbers [107], and an emphasis in government policy on environmental conservation rather than food production [59,106], meaning farmers have been rocked by the chop and change in farming schemes and are unsure of their role [64]. Some 37% of UK horticultural producers have been ‘unable to make plans’ [107], because of the uncertainty surrounding Brexit. This is also in the context of the COVID-19 pandemic. Nearly half of businesses in the study planned to ‘scale back’ production. Indeed, uncertainty does seem to be a common theme when discussing Brexit impacts on UK food security. Estimates suggest that of the 75,000 seasonal farm workers required annually in the UK, 98% have historically come from the EU [64,108]. Facing the loss of the free movement of seasonal and full-time workers to the UK from the EU, a benefit of being in a single market before Brexit, was/is a significant shock to the horticultural sector in the UK. This is particularly important in view of the UK’s low level of fresh fruit self-sufficiency. These dynamic and inter-relating politico-economic relationships are influencing extant and emerging food policy in the UK and affect food system resilience.

## 5. Discussion

Politico-economic forces drive food and public health policy, and three questions were posed in this research. Question 1 was “How are the politico-economic relationships around food affected by the implementation of Brexit, and then compounded by other recent shocks?” The implementation of Brexit and subsequent political and market shocks are affecting the politico-economic relationships around food. By recalibrating the trade relationships the UK has with other countries, and changing the support UK farmers receive, especially the delinking/ceasing of direct public payments in England, uncertain new policy and market environments are being created. Whilst the UK is embracing food supply neoliberalism, with more international trade and partnerships, arguably, an opportunity for safeguarding the importance of food as a public good, even a legal right, was missed in the scoping and implementation of recent UK legislation. The differentiation between the implementation of agricultural policy in the four home nations, the elements of the 2020 Agriculture Act, together with more recent legislation and the introduction of the Land Use Framework in England distanced land managers from primarily food provision as a public good, and they are instead being required to deliver multiple economic and societal benefits, where one of them is food production. As the effect of Brexit is not uniform across UK industrial sectors in terms of legal and economic uncertainty, migration processes, trade agreements, infrastructural and urban demands, and skills shortages could prove to be limiting factors, not only in land management but in other sectors too. For example, Ref. [109] examined the influence of Brexit on skills shortages in the UK construction industry, as was previously described in horticulture [107].

Question 2 was “Why are the paradigms influencing contemporary politico-economics framed around food price rather than national food security?” Treating food as a traded commodity creates a focus on financial value and extractive economic models rather than on more regenerative and circular economic options. Whilst regenerative approaches, an even distribution of financial value in the food supply chain, and the promotion of non-financial value, e.g., nutritional security, health, and well-being, are being focused on more, price is still a key driver of UK food markets, especially given the inflationary nature of food prices over the last few years. The UK Government has adopted interventions to ensure food security, either by increasing minimum wages, increasing taxation to support social interventions, or making food more available or accessible through imports via trade deals. However, there are concerns over the development of a ‘two-tier’ food system with UK-produced foods being destined for exports whilst lower-quality food is imported for the UK population to consume. The levels of non-communicable food-related disease in the UK [110] may impact Government spending in the future in terms of health, with costs associated with overweight and obesity alone currently estimated to reach £9.7 billion by 2050 [111], as it steers away from traditional neoliberal frameworks towards developing interventions that have aspects of neo-developmentalism. The dichotomy between recognizing the intrinsic right of citizens to adequate and nutritious food and the use of access to food supply to the UK as a politico-economic tool continues to evolve, and the consequences of a ‘two-tier food system’ [112] have yet to be fully understood.

Question 3 was “What forms of neoliberalism are emerging in the post-Brexit UK agri-food system?” The overriding paradox of the emerging UK form of neo-developmentalism is the clash between the idea of national self-determination and reducing regulatory control and rejecting free movement of labor, goods, and capital from the EU, and the idea that such free movement is also intrinsically neoliberal because it promotes both inter-country competition and capital-based growth, i.e., being within the EU and leaving the EU both have different and mutually exclusive neoliberal framings. New forms of neoliberalism are emerging that focus on policy interventions (Land Use Framework in England) to steer policy on future land use. However, the increasing demands for land in the UK (housing, infrastructure, carbon capture, nature recovery, etc.) suggests that a more extractive approach to natural capital is required, herein described as eco-extractivism. This form of eco-extractivism sees the ‘sustainable’ intensification of food production on some areas of land areas to release other land for other uses. Alternatively, another extractive neoliberal approach, eco-imperialism, drives the importing of food to then free up areas within the UK for land use, with associated impacts observed in those countries exporting food. The delinking of financial public support for land managers, and the financial gap in farm business income before proposed private carbon and biodiversity ‘markets’ emerge, creates a high degree of uncertainty for farmers who need to plan for their long-term future.

## 6. Concluding Thoughts

The politico-economic effects of Brexit have altered food-related relationships, recalibrating trade interactions and changing the public funding that UK farmers receive. Through this realignment of neoliberal positioning and economic models, especially if imported products are of a lower quality, there is greater potential for a ‘two-tier’ food system to emerge. A dichotomy exists between recognizing the intrinsic right of citizens to adequate and nutritious food and maintaining the existing cultural dynamics of UK food supply, and the use of agri-food policy as a politico-economic tool to drive higher economic growth within national boundaries and through trade deals. The role of Brexit itself in this realignment is not easy to establish because of the multiple politico-economic shocks that occurred almost simultaneously. Post-Brexit UK policy for food security and food safety appears to be one employing neo-developmentalism, especially the development of political and industrial strategies that reconfigure rural assets from their current focus on food production to deliver growth or renewal in other industrial sectors, such as house building, infrastructure, the delivery of net zero, and biodiversity recovery. The processes of state ‘interventions’ (legislation, policy, trade deal among others) that accelerate this realignment will have long-term implications for trade and could, if not appropriately mitigated, drive eco-imperialism and eco-extractivism, both at home and across the world, in an effort to attain national economic growth and meet supranational environmental targets.

## Figures and Tables

**Table 1:** Contemporary features of national and international forms of neoliberalism.

National Features	International Features
Against wealth distribution/redistribution at national level.	Free-market capitalism/pro-market global policy.
Emphasis on freedom, opportunities, and choice for individuals.	Foreign direct investment (FDI) and globalization.
Limited national government intervention.	Limits international governance and restrictions on trade and the movement of people across borders.
Supports austerity and reducing the cost of national government.	Supports reducing the cost of doing business for transnational corporations.
Supports entrepreneurship and innovation to drive national growth.	Supports international competition and allocation of activity to the countries that are economically ‘best’ to deliver the activity.
Supports privatization if it is the ‘best’ economic option.	Supports privatization if it is the ‘best’ economic option.

**Table 2:** Food security data (production to supply ratio) for the UK 2021 and 2023 (adapted from [49]).

Production to Supply Ratio Descriptor	2021	2023
All food	61%	62%
Indigenous food	74%	75%
Fresh fruit	15%	16%
Fresh vegetables	57%	53%
Cereal production	86%	93%
Beef	83%	85%
Lamb	108%	114%
Milk	105%	105%
Pork	71%	64%
Poultry meat	98%	82%
Poultry eggs	92%	97%

## Data Availability

No data were created in the development of this research.

## Abbreviations

The following abbreviations are used in this manuscript:

BSE	Bovine Spongiform Encephalopathy
CAP	Common Agricultural Policy
Defra	Department for Environment, Food and Rural Affairs
ESG	Environmental and Social Governance
EC	European Commission
EU	European Union
EU–US	European Union–United States
FAO	Food and Agriculture Organization
FAOSTAT	Food and Agriculture Organization Statistics
FTA	free trade agreement
ICESCR	International Covenant on Economic, Social and Cultural Rights
I-SDS	Investor–State Dispute Settlement
NFU	National Farmers’ Union
NZ	New Zealand
NZ–UK	New Zealand–United Kingdom
SDG	Sustainable Development Goal
TCA	Trade and Cooperation Agreement
T-TIP	Transatlantic Trade and Investment Partnership
UDHR	Universal Declaration of Human Rights
UK	United Kingdom
WTO	World Trade Organization
